# Recent advances in the treatment of renal stones using flexible ureteroscopys

**DOI:** 10.1097/JS9.0000000000001345

**Published:** 2024-03-12

**Authors:** Min He, Yonghui Dong, Wansong Cai, Jiale Cai, Yaming Xie, Mingke Yu, Changjiu Li, Liping Wen

**Affiliations:** a School of the Second Clinical Medical College, Zhejiang Chinese Medical University; b First People’s Hospital of Fuyang; c Department of Urology, Affiliated Hangzhou First People’s Hospital, School of Medicine, Westlake University; d General Surgery, Department of Hepatobiliary and Pancreatic Surgery and Minimal Invasive Surgery, Zhejiang Provincial People’s Hospital (Affiliated People’s Hospital), Hangzhou Medical College; e Zhejiang Provincial Hospital of Chinese Medicine, Hangzhou, Zhejiang Province, People’s Republic of China

**Keywords:** artificially intelligent FURS, kidney stone, negative pressure suction sheath, robot-assisted FURS, single-use flexible ureteroscopy, thulium fiber laser

## Abstract

Upper urinary tract stones are a common urological disease that can be treated by flexible ureteroscopy (FURS) through the natural urinary tract, in addition to extracorporeal shock wave lithotripsy and percutaneous nephrolithotomy. The advantages of FURS are less trauma, faster recovery, and fewer complications, while its disadvantages include poor results of lithotripsy and stone extraction when dealing with larger stones, and prolonged operation time. Over the last two decades, the emergence of new technologies such as FURS combined with negative pressure suction, robot-assisted FURS, and artificially intelligent FURS, coupled with improvements in laser technology (the use of thulium fiber lasers and the invention of single-use flexible ureteroscopes (su-fURS) suitable for primary level application, have significantly increased the global adoption of FURS. This surge in usage holds a promising future in clinical application, benefiting a growing number of patients with renal calculi. Accompanied by changes in technical concepts and therapeutic modalities, the scope of indications for FURS is broadening, positioning it as a potential primary choice for urolithiasis treatment in the future. This review outlines the progress in employing FURS for the treatment of renal calculi in order to generate insights for further research.

## Introduction

Highlights

- Flexible ureteroscopy (FURS) is a safe and effective procedure for treating renal stones.
- Thulium fiber lasers rises to become one of the best lithotripsy tools.
- The development of single-use flexible ureteroscopes addresses the main limitations of conventional reusable ureteroscopes, enabling the performance of ureteroscopic laser lithotripsy in primary hospitals.
- The emergence of new technologies such as FURS combined with negative pressure suction, robot-assisted FURS, and artificially intelligent FURS has further updated FURS.
- The scope of ureteroscopic laser lithotripsy applications is expanding.

Kidney stones are among the most common clinical disorders of the urinary tract, affecting ~10% of adults worldwide^1^. The main symptoms include pain, hematuria, back pain, and discomfort. If not treated in time, it can lead to disease progression, seriously affecting patient health, and quality of life^1^. With the development of endoscopic equipment and minimally invasive technology, flexible ureteroscopy (FURS) has the advantages of less trauma, quicker recovery, and fewer complications in the treatment of kidney stones compared with traditional extracorporeal shock wave lithotripsy and percutaneous nephrolithotripsy^2^. In recent years, emerging technologies such as FURS combined with negative pressure suction sheath, robot-assisted FURS, and artificially intelligent FURS have received increasing attention from scholars and patients and will play an important role in the future treatment of kidney stones.

## History of FURS and its development (Table 1)

**Table 1:** Development history of ureteroscopy.

Year	Researchers	Event
1912	Hugh Hampton Young	Incidental finding in a 2-month-old boy with a dilated ureter, allowing visualization of the renal calyces in the renal pelvis with a small cystoscope
1957	Curtiss, Hirschowitz	Created the first soft endoscopes through combining a large number of glass fiber payloads into a bundle and fusing these fibers together at the end so that they could move independently according to their lengths
1964	Marshall	Reported for the first time the use of flexible ureteroscope in urology to be able to visualize the entire urinary lumen and define the site where the stone is present
1977	Goodman	Invented ureteroscopy and added perfusion and working channels on this basis
1983	The Welch Allyn Company	Invented miniature image sensors, replacing fiberoptic guidance and ushering in the era of electronic endoscopy
1987	Bagley	Introduced flexible ureteroscopes with working channels
2006	Gyrus ACMI	Released the first digital ureteroscope
2011	Bansal	Used the first disposable flexible ureteroscope

The chance finding by Young^3^ in 1912 that a dilated ureter allowed visualization of calyces in the renal pelvis via a small cystoscope in a 2-month-old male infant stimulated the development of FURS. Marshall^4^ first reported the use of FURS in urology in 1964. The FURS, designed by Curtis *et al*.^5^, allows visualization of the entire urological lumen and identification of the stone’s location. In 1977, Goodman^6^ advanced the development and transformation of FURS by inventing ureteroscopy, and incorporating perfusion and working channels based on this innovation. In 1983, the Welch Allyn Company^7^ in the United States developed a miniature image sensor, marking a significant breakthrough in the history of endoscopy. This sensor superseded optical-fiber-guided imaging and introduced an era of electronic endoscopy. In 1987, Bagley^8^ introduced what is now known as a flexible ureteroscope with a working channel. In 2006, Gyrus ACMI^9^ released its first digital ureteroscope. In October 2015, Boston Scientific launched the LithoVue, a mirror body 9.5F, dual-turn, antitorsion device with digital clear imaging. This marked a significant milestone as the first single-use ureteroscope designed for access to the upper urinary tract^10^.

In summary, from the widespread application of traditional fiber optic FURS to the emergence of digital FURS, to the emergence of su-fURS, and now to the further improvement of FURS combined with negative pressure suction sheath, robot-assisted FURS, and artificial intelligence FURS^3,11^, both have further expanded the indications for ureteroscopic surgery, benefiting more patients with kidney stones.

## Application of ureteroscopy in renal calculi

### Holmium laser lithotripsy

The European Association of Urology (EAU)^12,13^ Guidelines on Urolithiasis propose flexible ureteral lithotripsy (FURL) as the first-line treatment option for stones 1–2 cm in the upper urinary tract and as an alternative option for those 2–4 cm in the upper urinary tract stones^14^. Indications (Table 2) for FURL include: a) X-ray negative renal stones (<2 cm) that are difficult to locate with ESWL; b) lower calyceal stones that remain after ESWL; c) embedded lower calyceal stones (<2 cm) that are inadequately treated by ESWL; d) cases involving extreme obesity, severe spinal deformity, ectopic kidneys combined with renal stones, and difficulty in establishing a channel for PNL; e) stones that are hard and not conducive to ESWL; and f) renal diverticulum calculi. The effectiveness of holmium laser lithotripsy (HLL) during ureteroscopy for upper urinary tract stones is well-established. HLL was notably more effective for small stones than for larger stones^15^. For kidney stones with a diameter ≥2 cm, HLL efficiency is low and staged surgery is often necessary to achieve complete removal^16,17^. PCNL is frequently the preferred approach for kidney stones with a diameter ≥2 cm^18,19^. Although the success rate for kidney stone removal is high, complications such as infection, substantial bleeding, hemothorax, pneumothorax, kidney damage, and intestinal harm may also occur^20–22^. The success of PCNL depends on several factors, including age, distance of percutaneous renal access, anatomical irregularities, extent of hydronephrosis, and various stone-associated factors such as stone burden, location, morphology, density, and number^22^. Therefore, according to the EAU guidelines, FURL is recommended in cases where there are contraindications to PCNL (such as an uncorrected risk of bleeding) or for patients who do not wish to undergo PCNL.

**Table 2:** Indications.

ESWL	FURS	PCNL
a) Diameters <20 mm, renal pelvis stones or upper and middle calyx stones	a) X-ray negative renal stones (<2 cm) that are difficult to locate with ESWL	a) Renal stones ≥2 cm, symptomatic renal calyces or diverticula, ESWL and FURS failed renal stones
b) Diameters <10 mm, lower calyceal stones	b) Lower calyceal stones that remain after ESWL	b) Special types of kidney stones, including pediatric kidney stones, isolated kidneys, horseshoe kidneys, transplanted kidneys combined with stones
c) Diameters 10–20 mm, lower calyceal stones, exclusion of unfavorable factors for ESWL such as small angled funnel-shaped renal pelvis angle, narrow low calyceal neck, narrow funnel-shaped renal pelvis, long skin-stone distance	c) Embedded lower calyceal stones (<2 cm) that are inadequately treated by ESWL	
d) Diameters 20–30 mm or surface area <500 mm^2^, Staghorn stone, excludes some cysteine staghorn stones and the majority of the stone body in the renal pelvis	d) Cases involving extreme obesity, severe spinal deformity, ectopic kidneys combined with renal stones, and difficulty in establishing a channel for PNL	
e) ESWL alone is not recommended for other complex staghorn-shaped kidney stones	e) Stones that are hard and not conducive to ESWL	
	f) Renal diverticulum calculi	

ESWL, extracorporeal shock wave lithotripsy; FURS, flexible ureteroscopy; PCNL, percutaneous nephrolithotomy.

The decision to intervene surgically for small renal stones (<2 cm) is related to stone characteristics, co-morbidities, patient renal anatomy, and patient selection^12^. Surgical intervention should be aggressively pursued in the following cases: 1. the presence of residual stones despite pharmacological treatment, 2. a high risk of hydronephrosis due to stone drop in isolated kidneys or renal calyces (more common in the mid-ureteral and ureteral junctions), and; 3. recurrent symptoms such as renal colic^23^. It has been suggested that urolithiasis is the most common cause of renal forniceal rupture (73%), whereas most cases are due to small stones (1–5 mm) in the distal ureter (61%)^24^. Therefore, lithotripsy surgery for renal stones <2 cm is required when above symptoms are present.

The 2022 edition of the ‘Guidelines for the Diagnosis and Treatment of Chinese Urology and Andrology Diseases’^25^ advises that PCNL is preferrable for kidney stones ≥2 cm, whereas ureteroscopy is a viable alternative. Nevertheless, as ureteroscopy technology, supplementary lithotripsy, and stone removal instruments continue to evolve and refine, the use of FURL has gradually become more widespread.

Fiber ureteroscopy combined with a holmium laser is a widely used clinical treatment for patients with kidney stones. In a randomized controlled study of 111 patients, Hao *et al*.^26^ determined that FURL accelerates postoperative recovery, reduces the incidence of adverse reactions, alleviates pain, and enhances the quality of life in patients with kidney stones measuring 2–3 cm, without significantly affecting urea nitrogen and serum creatinine levels. Yu *et al*. treated 113 pediatric patients with kidney stones >2 cm in size between June 2014 and October 2019 using two different surgical methods: PCNL and FURL. The stone-free rates of PCNL and FURL were 80.9% (34/42) and 79.7% (67/84), respectively (*P*=0.19), and the incidences of complications were 52.5% (21/40) and 27.4% (27/73), respectively (*P*=0.01). It can be concluded that FURL has almost the same stone clearance effect as PCNL in the treatment of pediatric renal calculi larger than 2 cm, and its safety is higher, which may shorten the postoperative hospital stay and reduce iatrogenic trauma^27^. However, one study^28^ investigating the management of kidney stones ranging from 2 to 4 cm showed that the PCNL group had a primary stone free rate (SFR) of 91.7%, which was significantly higher than the 74% SFR reported by the retrograde intrarenal surgery (RIRS) group (*P*=0.04). Regarding the cost of the procedure, an analysis of outcomes and costs after mini-percutaneous nephrolithotomy (mPCNL) or FURL for the treatment of 1–2 cm renal calculi, based on data from a prospective randomized clinical trial by Dutta *et al*.^29^ showed that the direct cost of mPCNL was higher than that of FURL (*P*<0.05). However, a retrospective analysis by Bagcioglu *et al*.^30^ suggested that the use of mPCNL is less expensive than FURL due to the additional processing and ancillary equipment required for FURL. Another study performed a cost-benefit analysis (CBA) and calculated a benefit-cost ratio (BCR), concluding that reusable ureteroscopes are cost-effective in centers with high case volumes^31^. However, comparative studies evaluating cost-effectiveness are scarce. The main problem with cost-effectiveness studies is that both the costs and benefits vary among different institutions and countries, necessitating careful and cautious interpretation.

Continuous saline infusion is essential for achieving a clear surgical field during HLL under ureteroscopy. If the drainage is not smooth, an increase in renal pressure (IRP) can develop^32^. Recent research indicates a strong link between intrarenal pressure (IRP) and intrarenal body temperature (IRT). IRP is contingent on inflow and outflow levels, whereas IRT relies on laser parameters and irrigation flow rates. Not all the laser energy emitted during high-power laser lithotripsy is applied to the stone. The remaining energy is absorbed by the water, which increases the IRT^32^. As previously stated, higher IRT levels are linked to elevated pulse rates^33^. Temperature can reach 60°C after 10 s of laser activation at 40W^34,35^. Hence, consistent monitoring is crucial for preventing surgical and infectious complications^36^. When the IRP exceeds 30 mmHg and persists for over 45 s, the postoperative infection rate increases 3.277 fold compared to an IRP of ≤30 mmHg, and development of systemic inflammatory response syndrome becomes more likely. Furthermore, an extended surgical duration increases the vulnerability of the renal pelvic mucosa, which in turn facilitates the entry of infectious materials into the bloodstream, ultimately causing sepsis.

When the stone load increased, numerous small fragments tended to cover the surface of the residual stone during lithotripsy. This results in decreased operator observation and makes it impossible for the laser to contact the residual stone directly. Consequently, lithotripsy efficiency was reduced, and the surgical time was significantly prolonged. Furthermore, continued pressurized perfusion and flushing may result in blood escaping from the mucosa of the renal pelvis and calyces, and a significant quantity of stone powder suspension can cause the flushing fluid to become cloudy, triggering a further decrease in visual clarity^37^. The blizzard phenomenon^38^, formed by stone fragments during surgery, can negatively affect the judgment of the visual field and the lithotripsy effect, ultimately leading to insufficient lithotripsy. Therefore, staged surgery may be a reasonable plan for improving the efficacy of flexible ureteroscope for kidney stones >2 cm.

The removal of stones after lithotripsy is a crucial factor to consider during ureteroscopic surgery. Surgery may involve the use of a stone basket to retrieve slightly larger stones. However, this repeated grasping process carries the risk of damaging the mucosa and prolonging surgical time. For smaller fragments, complete removal may not be possible, and there is a high risk of subsequent ureteral stone street development. The formation of stone streets is related to the following factors: (1) presence of large leftover stones, (2) damage or blockage of the ureter, (3) inability to extract stones, and (4) persistence of blood clots and discharge. Therefore, researchers have suggested^37^ utilizing a high-frequency (40–50 Hz), low-energy (0.2–0.4 J) holmium laser to pulverize stones as much as possible during surgery, resulting in stone fragments <2 mm, achieving self-discharge, decreasing treatment expenses, and enhancing patient contentment.

In summary, the combination of conventional ureteroscopy and HLL has a stone clearance rate equivalent to that of PCNL in the treatment of patients with stones smaller than 2 cm. However, there are also limitations to consider, particularly uncontrollable IRP and inadequate postoperative stone clearance rates for patients with stones larger than or equal to 2 cm.

### Endoscopic combined intrarenal surgery for large kidney stones

As previously mentioned, PCNL, which is recommended by the EAU guidelines, remains the first choice for renal stones larger than 2 cm in diameter. FURL can be considered if contraindications to PCN exist. For complex renal stones, PCNL usually requires the creation of two or more channels to improve SFR; however, as the number of channels increases, renal parenchymal injury is progressively aggravated, and the rate of complications such as bleeding is increased^39,40^. In recent years, to improve SFR and reduce complications associated with multichannel PCNL, some studies have proposed that PCNL combined with FURL is safe and effective in the treatment of kidney stones.

Gao *et al*.^41^ conducted a study on subgroups of patients with unilateral complex renal stones treated between March 2013 and December 2016. The study included 40 in the super-mini-PCNL group (SMP group), 55 in the FURL group, and 45 in the combined SMP and FURL group. The postoperative 3-day SFR in the SMP group, FURL group, and the combined group were 77.5, 78.2, and 97.8%, respectively (*P*=0.010). Intraoperative blood loss, operative time, and hospital stay were significantly lower in the combined group than in the SMP group but higher than in the FURL group. The conclusion was that SMP combined with FURL is an effective method for the treatment of complex renal calculi.

Similarly, Ding *et al*.^42^ retrospectively analyzed data from 92 patients treated with SMP combined with FURL (Group A) and 113 patients treated with FURL alone (Group B) between January 2018 and December 2022. The results revealed that the 3-day postoperative SFR was significantly higher in Group A (85.87%) compared to group B (72.57%) (*P*=0.021). The retreatment rate in group A ( 3.26%) was significantly lower than that of group B (10.62%) (*P*=0.044). The SFR at 3 months was higher in group A (94.57%) than in group B (90.27%) (*P*=0.254). Apart from the differences in operation time, 12 h and 24 h postoperative VAS scores, and complication rates, which were not statistically significant, the mean hemoglobin drop, 6 h postoperative VAS scores, and postoperative hospital stay were significantly higher in group A than in group B (*P*<0.05). Finally, it was concluded that SMP combined with FURL has the advantage of higher early SFR with no increased risk of complications compared to FURL in the treatment of complex kidney stones.

It has also been suggested that the combination of FURL and SMP is a minimally invasive, safe, and effective procedure for the removal of multiple kidney stones in selected cases, particularly pediatric patients with multiple kidney stones. No patients experienced severe postoperative bleeding or transfusion; discharge from the hospital occurred 2–5 days after the procedure, and imaging conducted 3 months later revealed no residual stones^43^.

## New equipment, new technology

### Thulium laser lithotripsy

The holmium laser is the ‘gold standard’ for laser lithotripsy, and the energy it generates can vaporize water between the end of the fiber and the stone, thereby crushing the stone with micro-blasts. TFL have emerged as the sharpest tools for stone crushing in recent years.

In a prospective study^44^ by Corrales and Traxer involving 50 cases treated with TFL for ureteral and renal stones, 200 μm and 150 μm TFL were used for ureteral and renal stones, respectively. Both groups demonstrated a low overall complication rate, leading to the conclusion that TFL is a safe and effective method for interstitial lithotripsy in RIRS. In another study by Vaddi *et al*.^45^, 126 patients treated with a 60W TFL developed hematuria in 12 cases and fever in 9 cases, which were Clavien class 1–2. The overall SFR was 93.6% and the complication rate was 16.6% (21/126). Ultrapulsed TFL was equally efficient and safe.

In comparison to HLL, Chua *et al*.^46^ found no statistically significant difference in SFR between groups in 1698 cases (RR 1.09, 95% CI: 0.99–1.20). However, when compared with a subgroup analysis of holmium lasers without pulsed modulation, SFR was superior for TFL (RR 1. 11, 95% CI: 1.01–1.23), and operative time (OT), laser usage time (LUT), and ablation rate (stone volume/laser time) were significantly better with TFL than with HLL (SMD −1.19, 95% CI: −1.85 to −0.52; SMD −1.67, 95% CI: −2.62 to −0.72; SMD 0.59, 95% CI: 0.15–1.03; respectively). Conversely, TFL demonstrated a significantly lower degree of backlash compared to HLL without pulse modulation (SMD −1.23, 95% CI: −1.74 to −0.71).

The authors converted electrical energy into light energy with higher conversion efficiency and better beam quality compared to holmium lasers. The generated laser beam is more uniform, has better focusability, and can be transported through finer optical fibers, providing a larger perfusion space, wider pulse repetition frequency range, and lower pulse energy. The superpulse mode significantly improves the efficiency of stone ablation and reduces the incidence of stone regression. In addition, it has a shallow tissue penetration depth and good hemostatic performance. Therefore, TFL may replace holmium lasers in the future due to these advantages^47–50^. However, the thulium laser has some shortcomings; with a wavelength of 1950 nm, it has a higher water absorption coefficient, higher ablation efficiency, and higher energy absorption. Nevertheless, this higher rate of energy transfer to the stone and surrounding fluid can result in thermal damage to the uroepithelium^51^. Sierra *et al*. conducted an in vitro study on thermal damage and laser efficiency of TFL and concluded that thermal damage increased progressively with rising output energy. More thermal damage was observed with less experienced operators in a high-frequency environment^52^. Both the holmium and TFL produced elevated ureteral fluid temperatures in a series of tests performed in clinical power settings. Within 60 s of continuous laser activation, the thulium laser produced significantly higher mean ureteral fluid temperatures than the holmium laser at all power levels tested (*P*<0.001). At 30 W, the thulium laser was 11% warmer than the holmium laser, exceeding 43°C^51,53^.

### Single-use flexible ureteroscopes

In recent years, su-fURS and semi-rigid ureteroscopes have been developed as alternatives to reusable ureteroscopes. These disposable devices aim to address the issues associated with reusable ureteroscopes, including the substantial expenses related to the procurement, sterilization, handling, upkeep, and repair of instruments^54^. By eliminating the need to disinfect optical cables, the likelihood of maintenance-induced and sterilization-induced damage is diminished, resulting in a decreased probability of infections related to device use.

Marchini *et al*.^55^ demonstrated that, regarding usage, SFR and complications were comparable between disposable and reusable ureteroscopes. Bragaru *et al*.^56^ supported this by noting that disposable fURS is as effective in treating kidney stones as reusable fURS. Goger *et al*.^57^ revealed that clinical effectiveness and complication rates were comparable when utilizing RIRS for the treatment of renal calyceal stones. Bragaru *et al*.^58^ further demonstrated that the SFR for three types of su-fURS, namely, Uscope 3022, LithoVue, and EU scope, is marginally superior to that of patients who typically undergo reusable fURS treatment. Moreover, Patil^59,60^ suggested that various types of sufURS exhibit similar clinical performance. At a particular distance, all the fURS provided equivalent image resolution and power. The irrigation flow of all scopes was uniform in both the deflected and undeflected states when the working channel was empty.

Despite these advantages, sufURS has gained popularity among urologists. However, some scholars argue that^61^ while sufURS may address the limitations of reusable ureteroscopes, it high cost and environmental concerns require further investigation and resolution before routine implementation. Taguchi *et al*.^62^ conducted a micro-cost comparison between a reusable flexible ureteroscope (URF-P6) and LithoVue, with a total cost per ureteroscope of $2799.72 and $2852.29 for the URF-P6 and LithoVue, respectively. The cost of repairing flexible ureteroscopes varies depending on the model and type of breakage. According to Kramolowsky *et al*., the total cost per repair for the URF-P6 (Olympus) ranged from $233 to $7521, with an average cost of $355 per case. For the URF-V series (Olympus), the total maintenance cost was $119 632, with an average cost per box of $511^63^. In a prospective cohort study, reusable ureteroscopy costs were $1212 and $1743, compared to $1300 to $3180 for single-use ureteroscopy procedures^64^. To date, only one study has specifically evaluated the environmental life cycle of the LithoVue versus the Olympus Flexible Video Ureteroscope (URV-F). Davis *et al*.^65^ concluded that, overall, the LithoVue (4.43 kg) has a comparable carbon footprint to the URV-F (4.47 kg), without considering factors such as natural resources, greenhouse gas emissions from incineration, and landfill waste.

It has been suggested that su-fURS may be used to preserve ru-fURS in complex urological cases with a high risk of endoscopic injury or fracture. The use of su-fURS may be influenced by the volume of the procedure, the limited availability of sterilization equipment, the durability of ru-fURS, and the potential risk of infectious complications^66^. Adoption of a standardized technology known as ureteroscopic HLL is widespread in the field of urology in China. However, most urology departments offer a limited number of reusable ureteroscopes, necessitating prompt disinfection after each use. This requires a meticulous level of disinfection and waiting for an appropriate disinfection time. In cases of equipment damage, ureteroscope repair requires an extended period. Hence, the scenario of ‘supply not meeting demand’ is certain to arise, leading to a shortage in procurement at high costs. Subsequently, this impact the ability of community hospitals to perform surgeries. Nevertheless, the advent of su-fURS presents an opportunity to redress the situation, potentially enabling the rational allocation of medical resources and yielding enhanced benefits to a larger patient population.

### Combined negative pressure suction sheath

To address the issue of elevated pressure in the renal pelvis during ureteroscopic lithotripsy and the inclination of leftover stone fragments to generate stone streets postoperatively, negative-pressure suction technology has been integrated with ureteroscopic lithotripsy.

Deng *et al*.^67^ previously developed a UAS for the ureteral pathway equipped with a suction and perfusion platform and a pressure-sensitive tip. Using advanced computer technology, the system records and monitors real-time RPP, allowing for accurate adjustment of the perfusion flow rate and vacuum suction. The authors observed that the actual IRP remained <30 mmHg, resulting in a clear intraoperative field of view. Postoperative SFR was 90.0 and 95.6% at 1 month. The automated RPP control makes this system safe and effective for treating kidney stones.

De Coninck *et al*.^68^ colleagues previously proposed that maintaining a ureteroscopy-to-UAS diameter ratio of less than 0.75 can keep IRP below the renal pelvic venous reflux threshold (40–60 cmH2O) when the pressure surpasses 200 cmH2O during forced perfusion.

One prior study^69^ showed that when utilizing negative pressure suction devices alongside FURS and PCNL for treating solitary kidney stones measuring 2–3 cm, the FURS group showed a shorter hospital stay than the PCNL group. Furthermore, the PCNL group required more painkillers than the FURS group. Nevertheless, there was no significant difference in the mean surgical duration or stone clearance between the two groups. Both surgical techniques are equally safe and efficient. However, FURS is superior to RIRS in terms of length of hospital stay, occurrence of complications, and blood loss.

A study conducted by Zewu *et al*.^70^ found that using a combination of ureteroscopy and a negative-pressure suction sheath in the treatment of kidney stones resulted in a higher timely SFR, shorter surgical time, and lower incidence of infectious complications compared to using an ordinary sheath. Similarly, Huang *et al*. enrolled 371 patients who had received successful fURL treatment in their study. These patients were subsequently categorized into two distinct groups: the traditional fURL group and the vacuum-assisted dedusting lithotripsy (VADL) group. After performing a 1:1 propensity score matching analysis to compare the outcomes of the two groups, a stratified analysis was performed based on stone size and location. The results showed that the use of VADL technology can significantly enhance postoperative SFR in patients with renal stones or stones <3 cm in the proximal ureter treated with ureteroscopy^71^.

A safe and efficient device, the omnidirectional (OD) ureteral access sheath (UAS), was first described by Jie *et al*.^72^ in their recent study. The OD UAS had a deflection angle of ~110–130° when not loaded, and 90–130° when loaded with various instruments. The distinctive pliable construction of the device enables it to navigate the renal calyces and locate stones in conjunction with a flexible ureteral endoscope. The OD UAS connects to the suction port, improves the single SFR, and simplifies high-load kidney stone treatment, while reducing the operative duration.

**Figure 1:**
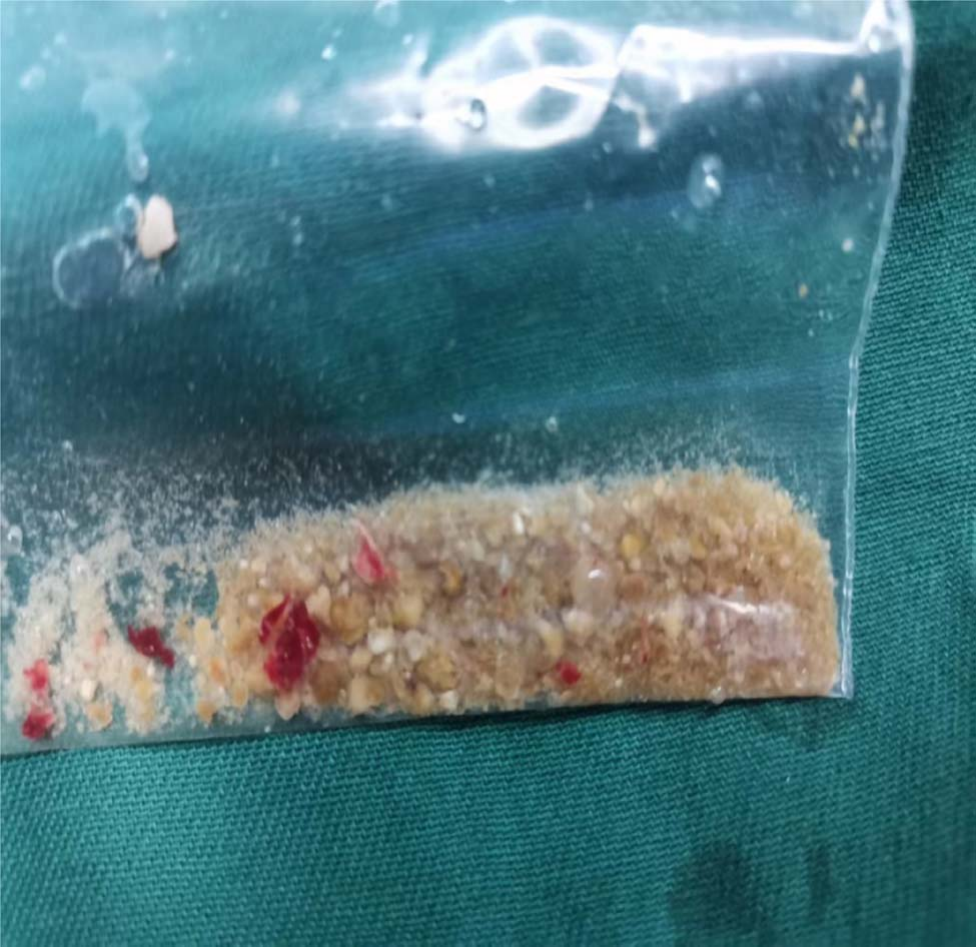
Ureteroscopy combined with negative pressure suction for holmium laser lithotripsy for extracting stone fragments after surgery.

The technique of combining ureteroscopy with negative pressure suction HLL is highly effective for pulverizing even large kidney stones (Fig. 1). This approach not only improves the overall lithotripsy efficiency but also reduces the incidence of stone streets, thus allowing for a wider range of indications for lithotripsy. However, the placement of a negative pressure suction sheath as a ureteral access sheath (UAS) increases the risk of ureteral wall ischemia and injury to the ureteral mucosa or muscularis propria, which theoretically increases the risk of ureteral stricture^73^. There is also a disadvantage related to difficulty in dealing with infrarenal calyx stones due to the restricted bending angle and postoperative pain^74^.

### Robot-assisted FURS, artificial intelligence FURS

In 2008, researchers used the Sensei Magellan System (designed specifically for interventional cardiology) FURS robot for 18 clinical trials. However, it was discontinued due to its ability to passively manipulate the ureteroscope^11^. The Roboflex Avicenna, designed specifically for FURS, consists of a manipulator to operate the ureteroscope and a surgeon’s console. This console facilitates fine adjustment of the scope, automated insertion and removal, and repositioning establishing a suitable and safe platform for robotic FURS with significantly improved ergonomics. The surgeon can perform stable and precise surgical operations while seated at the open console, as well as laser techniques and fluoroscopy via the touch screen and foot pedals, significantly increasing efficiency and comfort while reducing the surgeon’s exposure to radiation^75–78^.

In a study by Chen *et al*. in which AI-controlled pressure FURS and MPCNL were used for the treatment of 2–3 cm isolated renal stones, AI-controlled pressure group exhibited advantages such as a shorter hospital stay [(3.53±1.25) d<(6.54±2.36) d] and fewer complications. In addition to pressure control, in lithotripsy, the AI can preset the laser lithotripsy program, set the optimal parameters, and blast with extreme speed for instantaneous lithotripsy^69^. Recent research reports the emergence of a new FURS robot with a force feedback function. Using a neural network-based approach to estimate the interaction force between the flexible ureteral scope and the environment, the operator can accurately perceive obstacles when the interacting axial force or torque exceeds 1.2 N or 15.6 mN·m, respectively, during the robot’s teleoperation of the flexible ureteral scope. This force feedback greatly improves the accuracy of the operator’s obstacle perception and is expected to improve the safety of robotic-assisted FURS^79^.

## Complications and management methods after ureteroscopic surgery

After more than half a century of development and enhancement, ureteroscopic lithotripsy has evolved into a minimally invasive procedure^23^. However, postoperative complications remain a significant problem^80^, and may include: 1. Surgical injuries such as submucosal false channel, mucosal injuries, ureteral perforations, and ureteral avulsions. 2. Complications related to perfusion pressure, including infection, urinary sepsis, bleeding, perirenal fluid accumulation, and renal rupture. 3. Issues related to ureteral stents, including improper stent placement and symptoms related to ureteral stents. 4. Others, such as postoperative bladder ureteral reflux and long-term ureteral stenosis. A careful and expert lithotripsy process can markedly reduce the postoperative complications associated with surgical injuries. Most submucosal false channel can be successfully restored by implanting a stent that remains for 2 weeks (crossing the injury plane), or by a direct visual operation that effectively minimizes the injury. For example, in cases of bleeding, accumulation of fluid around the kidney, and rupture of the kidney, most patients can be treated conservatively (bed rest, blood transfusion, etc.) and recover without requiring surgical intervention. Nevertheless, if required, perirenal drainage and interventional treatment may be considered, with the possibility of nephrectomy^81,82^. The incidence of stent-related symptoms is high and has a significant impact on patient quality of life. A large number of studies have confirmed that M-receptor blockers and α-receptor blockers can effectively alleviate related symptoms^83^, but there is still controversy over whether the combination of the two has a greater effect than one plus one.

It should be noted that the overall complication rate after FURL is 9–25%^13^. Mostly postoperative infections such as urinary tract infections (UTI), urosepsis (severe infections), etc. The incidence of urosepsis ranges from 0.5 to 11.1% and that of septic shock from 0.3 to 4.6%^84^. However, given the seriousness and life-threatening nature of urosepsis, it is important to understand its potential risk factors.

In a retrospective study, Baboudjian *et al*.^85^ analyzed data from 604 patients who underwent FURL at their hospital between January 2015 and March 2019. On multivariate analysis, postoperative UTI was found to be associated with female sex [OR 2.20 (1.02–5.02), *P*=0.04], UTI in the previous 6 months [OR 2.34 (1.12–5.11), *P*=0.02], and increased duration of surgery [OR 1.02 (1.002–1.031), *P*=0.02].

Bhojani *et al*.^86^ conducted a systematic search of Medline, Embase, and the Cochrane Central Register of Controlled Trials for studies on risk factors for postoperative urosepsis after FURL. The incidence of postoperative urosepsis was 5.0% (95% CI: 2.4–8.2) in 5597 patients across 13 studies, including 5 prospective studies. Older age (mean difference=2.7 years, *P*=0.002, six studies), diabetes mellitus (odds ratio=2.04, *P*=0.04, six studies), ischemic heart disease (odds ratio=2.49, *P*=0.002, two studies), preoperative stenting (odds ratio=3.94, *P*<0.001, six studies), and positive urine culture (odds ratio=3.56, *P*<0.001, six studies), and longer operative time (mean difference=9 min, *P*=0.02, one study) were associated with an increased risk of postoperative UTI.

In addition, Sun *et al*.^87^ concluded that postoperative infectious complications were associated with female sex, diabetes mellitus, preoperative and postoperative stent placement, and longer duration of surgery. However, a positive preoperative urine culture was the most important risk factor (OR 2.95, 95% CI: 1.96–4.43, *P*<0.01). Therefore, patients with positive urine cultures should receive adequate preoperative antibiotic treatment based on the results of drug susceptibility testing. Patients with negative urine cultures should receive prophylactic antibiotic treatment once on the day of surgery, while a ureteroscopic sheath should be used during surgery to improve intraoperative drainage, control perfusion pressure, and control hand surgery time, which can effectively reduce the risk of postoperative infection. Surgical treatment may be staged in cases with high loads and infectious stones. For patients who have already developed urinary sepsis, it is best to use potent antibiotics within 1 h of onset, actively maintain circulatory stability, maintain water-electrolyte and acid-base balance, support the use of glucocorticoids, and, if necessary, use fresh frozen plasma and other blood products to prevent disseminated intravascular coagulation (DIC). In recent years, clinical trials have confirmed the efficacy of bundle therapy in treating septic shock. Implementing bundle therapy for early infectious septic shock has proven effective in improving medical staff compliance with septic shock treatment, enhancing patient blood circulatory function, and decreasing patient mortality rates. Not only does it have significant clinical practical value, but it can also substantially lower medical expenses and ease the burden of treatment costs on the families of patients^88–90^.

## Outlook and conclusion

In summary, the scope for the application of ureteroscopy for the treatment of kidney stones is constantly expanding due to the ongoing development of ureteroscopy technology, frequent updates to lithotripsy auxiliary equipment, and the continuous improvement of lithotripsy lasers. In the future, Roquero *et al*. aims to enhance ureteroscopic surgery using the stone debris removal system (MagSToNE). This system consists of magnetic hydrogel, magnetized stone fragments, and a magnetic wire to remove the fragments. Additionally, In the future, RIRS may be an alternative therapy to PCNL, with acceptable efficacy and complication rates for renal stones^91^. The negative-pressure suction sheaths play an important role in the treatment of kidney stones with a diameter ≥2 cm by maintaining an appropriate level of intraoperative renal pelvic pressure, maintaining a clear field of view during surgery, improving stone removal efficiency, accelerating stone removal speed, shortening surgical time, and reducing postoperative complications such as infection and bleeding. TFL are thinner, allowing more room for maneuver, and its ultrapulsed mode of operation significantly improves stone ablation efficiency while reducing the incidence of stone regression. Disposable ureteroscopy can reduce the incidence of instrument-related infections, allowing primary lithotripsy to be performed. Currently, there is very little research on the treatment of kidney stones using disposable ureteroscopy combined with negative-pressure suction and sheath thulium laser. We believe that the combination of the three could bring good news to patients with kidney stones by reducing trauma, alleviating pain, and accelerating recovery.

In the foreseeable future, the application of Roquero *et al*.’s stone removal system (MagSToNE), which incorporates a magnetic hydrogel for wrapping and magnetizing stone fragments and a magnetic wire for removing the fragments), as well as the advent of ultra-fine flexible endoscopes and lithotripsy robots, will further advance ureteral flexible endoscopy. As urology transitions into the era of minimally invasive and intelligent surgery, multidisciplinary associations such as artificial intelligence and medical-industrial crossover may present the future trend of FURS development. Simultaneously, an increasing number of urologists will become proficient in the use of FURS through the use of 3D printing technology and virtual reality simulators with realistic visual effects, which will increase the enjoyment of training and keep training costs reasonable^92–94^.

## Ethical approval

Not applicable.

## Consent

Not applicable

## Source of funding

None.

## Author contribution

M.H.: conceptualization, writing – original draft, and writing – review and editing; Y.D.: conceptualization and writing – review and editing; W.C.: writing – review and editing; J.C. and Y.X.: software and writing – review and editing; M.Y.: writing – review and editing and investigation; C.L.: writing – review and editing, supervision, and project administration; L.W.: conceptualization, supervision, and project administration.

## Conflicts of interest disclosure

The authors have no potential conflicts of interest to disclose.

## Research registration unique identifying number (UIN)

Not applicable.

## Guarantor

Liping Wen, First People’s Hospital of Fuyang, Hangzhou 311400, Zhejiang Province, People’s Republic of China. E-mail: winleopard@163.com. Changjiu Li, Affiliated Hangzhou First People’s Hospital, School of Medicine, Westlake University, Hangzhou 310006, Zhejiang Province, People’s Republic of China. E-mail: li_changjiu@126.com.

## Data availability statement

Not applicable.

## Provenance and peer review

Not commissioned, externally peer-reviewed.

## References

[1] Singh P Harris PC Sas DJ . The genetics of kidney stone disease and nephrocalcinosis. Nat Rev Nephrol 2022;18:224–240. 34907378 10.1038/s41581-021-00513-4

[2] Doizi S Traxer O . Flexible ureteroscopy: technique, tips and tricks. Urolithiasis 2018;46:47–58. 29222575 10.1007/s00240-017-1030-x

[3] Reis Santos JM . Ureteroscopy from the recent past to the near future. Urolithiasis 2018;46:31–37. 29188308 10.1007/s00240-017-1016-8

[4] Marshall VF . Fiber optics in urology. J Urol 1964;91:110–114. 14106571 10.1016/S0022-5347(17)64066-7

[5] Hirschowitz BI Peters CW Curtiss LE . Preliminary report on a long fiberscope for examination of stomach and duodenum. Med Bull (Ann Arbor) 1957;23:178–180. 13433948

[6] Pearle MS Calhoun EA Curhan GC . Urologic diseases in America project: urolithiasis. J Urol 2005;173:848–857. 15711292 10.1097/01.ju.0000152082.14384.d7

[7] Demling L Hagel HJ . Video endoscopy. Fundamentals and problems. Endoscopy 1985;17:167–169. 4054060 10.1055/s-2007-1018491

[8] Bagley DH Huffman JL Lyon ES . Flexible ureteropyeloscopy: diagnosis and treatment in the upper urinary tract. J Urol 1987;138:280–285. 3599238 10.1016/s0022-5347(17)43119-3

[9] Gridley CM Knudsen BE . Digital ureteroscopes: technology update. Res Rep Urol 2017;9:19–25. 28203551 10.2147/RRU.S104229 PMC5293503

[10] Butticè S Sener TE Netsch C . LithoVue™: a new single-use digital flexible ureteroscope. Cent European J Urol 2016;69:302–305. 10.5173/ceju.2016.872 PMC5057057 27730000

[11] Rassweiler J Fiedler M Charalampogiannis N . Robot-assisted flexible ureteroscopy: an update. Urolithiasis 2018;46:69–77. 29170856 10.1007/s00240-017-1024-8

[12] Geraghty RM Davis NF Tzelves L . Best practice in interventional management of urolithiasis: an update from the European Association of Urology Guidelines Panel for Urolithiasis 2022. Eur Urol Focus 2023;9:199–208. 35927160 10.1016/j.euf.2022.06.014

[13] Türk C Petřík A Sarica K . EAU guidelines on interventional treatment for urolithiasis. Eur Urol 2016;69:475–482. 26344917 10.1016/j.eururo.2015.07.041

[14] Zeng G Traxer O Zhong W . International Alliance of Urolithiasis guideline on retrograde intrarenal surgery. BJU Int 2023;131:153–164. 35733358 10.1111/bju.15836 PMC10084014

[15] Mi Y Ren K Pan H . Flexible ureterorenoscopy (F-URS) with holmium laser versus extracorporeal shock wave lithotripsy (ESWL) for treatment of renal stone <2 cm: a meta-analysis. Urolithiasis 2016;44:353–365. 26530230 10.1007/s00240-015-0832-y

[16] Hyams ES Munver R Bird VG . Flexible ureterorenoscopy and holmium laser lithotripsy for the management of renal stone burdens that measure 2 to 3 cm: a multi-institutional experience. J Endourol 2010;24:1583–1588. 20629566 10.1089/end.2009.0629

[17] Cohen J Cohen S Grasso M . Ureteropyeloscopic treatment of large, complex intrarenal and proximal ureteral calculi. BJU Int 2013;111(3 Pt B):E127–E131. 22757752 10.1111/j.1464-410X.2012.11352.x

[18] Kim BS . Recent advancement or less invasive treatment of percutaneous nephrolithotomy. Korean J Urol 2015;56:614–623. 26366273 10.4111/kju.2015.56.9.614 PMC4565895

[19] Desai M Sun Y Buchholz N . Treatment selection for urolithiasis: percutaneous nephrolithomy, ureteroscopy, shock wave lithotripsy, and active monitoring. World J Urol 2017;35:1395–1399. 28303335 10.1007/s00345-017-2030-8

[20] Grosso AA Sessa F Campi R . Intraoperative and postoperative surgical complications after ureteroscopy, retrograde intrarenal surgery, and percutaneous nephrolithotomy: a systematic review. Minerva Urol Nephrol 2021;73:309–332. 33887891 10.23736/S2724-6051.21.04294-4

[21] Kyriazis I Panagopoulos V Kallidonis P . Complications in percutaneous nephrolithotomy. World J Urol 2015;33:1069–1077. 25218854 10.1007/s00345-014-1400-8

[22] Kallidonis P Panagopoulos V Kyriazis I . Complications of percutaneous nephrolithotomy: classification, management, and prevention. Curr Opin Urol 2016;26:88–94. 26555687 10.1097/MOU.0000000000000232

[23] Türk C Petřík A Sarica K . EAU guidelines on diagnosis and conservative management of urolithiasis. Eur Urol 2016;69:468–474. 26318710 10.1016/j.eururo.2015.07.040

[24] Morgan TN Bandari J Shahait M . Renal forniceal rupture: is conservative management safe? Urology 2017;109:51–54. 28801219 10.1016/j.urology.2017.07.045

[25] Huang J Zhang X . Guidelines for the Diagnosis and Treatment of Chinese Urology and Andrology Diseases. Science Press; 2022:p413–p456.

[26] Hao X Li C Dun W . Effect of flexible ureteroscopic lithotripsy on surgical outcomes, renal function and quality of life of patients with 2-3 cm renal calculi. Arch Esp Urol 2023;76:189–195. 37340524 10.56434/j.arch.esp.urol.20237603.22

[27] Zhang Y Li J Jiao JW . Comparative outcomes of flexible ureteroscopy and mini-percutaneous nephrolithotomy for pediatric kidney stones larger than 2 cm. Int J Urol 2021;28:650–655. 33754401 10.1111/iju.14532

[28] Atis G Culpan M Pelit ES . Comparison of percutaneous nephrolithotomy and retrograde intrarenal surgery in treating 20-40 mm renal stones. Urol J 2017;14:2995–2999. 28299761

[29] Dutta R Mithal P Klein I . Outcomes and costs following mini-percutaneous nephrolithotomy or flexible ureteroscopic lithotripsy for 1-2-cm renal stones: data from a prospective, randomized clinical trial. J Urol 2023;209:1151–1158. 37157794 10.1097/JU.0000000000003397

[30] Bagcioglu M Demir A Sulhan H . Comparison of flexible ureteroscopy and micropercutaneous nephrolithotomy in terms of cost-effectiveness: analysis of 111 procedures. Urolithiasis 2016;44:339–344. 26474768 10.1007/s00240-015-0828-7

[31] Sarica K Yuruk E . Re: the economic implications of a reusable flexible digital ureteroscope: a cost-benefit analysis. Eur Urol 2017;72:652–653. 10.1016/j.eururo.2017.05.047 28625843

[32] Pauchard F Ventimiglia E Corrales M . A practical guide for intra-renal temperature and pressure management during rirs: what is the evidence telling us. J Clin Med 2022;11:3429. 35743499 10.3390/jcm11123429 PMC9224584

[33] Aldoukhi AH Dau JJ Majdalany SE . Patterns of laser activation during ureteroscopic lithotripsy: effects on caliceal fluid temperature and thermal dose. J Endourol 2021;35:1217–1222. 33397188 10.1089/end.2020.1067 PMC8670574

[34] Aldoukhi AH Ghani KR Hall TL . Thermal Response to High-Power Holmium Laser Lithotripsy. J Endourol 2017;31:1308–1312. 29048216 10.1089/end.2017.0679

[35] Lopes ACN Dall’Aqua V Carrera RV . Intra-renal pressure and temperature during ureteroscopy: Does it matter? Int Braz J Urol 2021;47:436–442. 33284547 10.1590/S1677-5538.IBJU.2020.0428 PMC7857755

[36] Panthier F Pauchard F Traxer O . Retrograde intra renal surgery and safety: pressure and temperature. A systematic review. Curr Opin Urol 2023;33:308–317. 37140545 10.1097/MOU.0000000000001102

[37] Patel AP Knudsen BE . Optimizing use of the holmium:YAG laser for surgical management of urinary lithiasis. Curr Urol Rep 2014;15:397. 24532341 10.1007/s11934-014-0397-2

[38] Keller EX De Coninck V Doizi S . What is the exact definition of stone dust? An in vitro evaluation. World J Urol 2021;39:187–194. 32270283 10.1007/s00345-020-03178-z

[39] Wu J He S Wang H . Efficacy and economy of two-stage percutaneous nephrolithotomy for complex renal calculi. Arch Esp Urol 2022;75:862–866. 36651097 10.56434/j.arch.esp.urol.20227510.125

[40] Wang Z Feng D Cao D . Comparison of safety and efficacy between single-tract and multiple-tract percutaneous nephrolithotomy treatment of complex renal calculi: a systematic review and meta-analysis. Minerva Urol Nephrol 2021;73:731–738. 33781020 10.23736/S2724-6051.21.04239-9

[41] Gao H Zhang H Wang Y . Treatment of complex renal calculi by digital flexible ureterorenoscopy combined with single-tract super-mini percutaneous nephrolithotomy in prone position: a retrospective cohort study. Med Sci Monit 2019;25:5878–5885. 31389405 10.12659/MSM.915034 PMC6693367

[42] Ding Q Zhu H Fan Z . Comparative analysis of super-mini percutaneous nephrolithotomy combined with flexible ureteroscopic lithotripsy versus flexible ureteroscopic lithotripsy alone for treating complex kidney stones: a retrospective study of 205 patients. Med Sci Monit 2023;29:e941012. 37994010 10.12659/MSM.941012 PMC10683530

[43] Li J Wang W Du Y . Combined use of flexible ureteroscopic lithotripsy with micro-percutaneous nephrolithotomy in pediatric multiple kidney stones. J Pediatr Urol 2018;14:281.e281–281.e286. 10.1016/j.jpurol.2018.03.005 29625868

[44] Corrales M Traxer O . Initial clinical experience with the new thulium fiber laser: first 50 cases. World J Urol 2021;39:3945–3950. 33590280 10.1007/s00345-021-03616-6

[45] Vaddi CM Ramakrishna P Ganeshan S . The clinical efficiency and safety of 60W superpulse thulium fiber laser in retrograde intrarenal surgery. Indian J Urol 2022;38:191–196. 35983111 10.4103/iju.iju_60_22 PMC9380467

[46] Chua ME Bobrowski A Ahmad I . Thulium fibre laser vs holmium: yttrium-aluminium-garnet laser lithotripsy for urolithiasis: meta-analysis of clinical studies. BJU Int 2023;131:383–394. 36260370 10.1111/bju.15921

[47] Enikeev D Herrmann TRW Taratkin M . Thulium fiber laser in endourology: current clinical evidence. Curr Opin Urol 2023;33:95–107. 36710593 10.1097/MOU.0000000000001057

[48] Terry RS Whelan PS Lipkin ME . New devices for kidney stone management. Curr Opin Urol 2020;30:144–148. 31895890 10.1097/MOU.0000000000000710

[49] Traxer O Keller EX . Thulium fiber laser: the new player for kidney stone treatment? A comparison with Holmium:YAG laser. World J Urol 2020;38:1883–1894. 30729311 10.1007/s00345-019-02654-5 PMC7363731

[50] Kronenberg P Traxer O . The laser of the future: reality and expectations about the new thulium fiber laser-a systematic review. Transl Androl Urol 2019;8(suppl 4):S398–s417. 31656746 10.21037/tau.2019.08.01 PMC6790412

[51] Belle JD Chen R Srikureja N . Does the novel thulium fiber laser have a higher risk of urothelial thermal injury than the conventional holmium laser in an in vitro study? J Endourol 2022;36:1249–1254. 35302382 10.1089/end.2021.0842

[52] Sierra A Corrales M Kolvatzis M . Thermal injury and laser efficiency with holmium YAG and thulium fiber laser-an in vitro study. J Endourol 2022;36:1599–1606. 35793107 10.1089/end.2022.0216

[53] Ortner G Rice P Nagele U . Tissue thermal effect during lithotripsy and tissue ablation in endourology: a systematic review of experimental studies comparing Holmium and Thulium lasers. World J Urol 2023;41:1–12. 36515722 10.1007/s00345-022-04242-6

[54] Scotland KB Chan JYH Chew BH . Single-use flexible ureteroscopes: how do they compare with reusable ureteroscopes? J Endourol 2019;33:71–78. 30612446 10.1089/end.2018.0785

[55] Marchini GS Torricelli FC Batagello CA . A comprehensive literature-based equation to compare cost-effectiveness of a flexible ureteroscopy program with single-use versus reusable devices. Int Braz J Urol 2019;45:658–670. 31397987 10.1590/S1677-5538.IBJU.2018.0880 PMC6837614

[56] Bragaru M Multescu R Georgescu D . Single-use versus conventional reusable flexible ureteroscopes - an evaluation of the functional parameters. J Med Life 2023;16:10–15. 36873117 10.25122/jml-2022-0269 PMC9979166

[57] Göger YE Özkent MS Kilinç MT . Efficiency of retrograde intrarenal surgery in lower pole stones: disposable flexible ureterorenoscope or reusable flexible ureterorenoscope? World J Urol 2021;39:3643–3650. 33738574 10.1007/s00345-021-03656-y

[58] Salvadó JA Elorrieta V Cabello JM . Clinical comparison between three single-use flexible ureteroscope models: a real-world experience. Urol Int 2022;106:1220–1225. 36318885 10.1159/000527179

[59] Patil A Agrawal S Singh A . A Single-center prospective comparative study of two single-use flexible ureteroscopes: lithovue (Boston Scientific, USA) and Uscope PU3022a (Zhuhai Pusen, China). J Endourol 2021;35:274–278. 32967450 10.1089/end.2020.0409

[60] Patil A Agrawal S Batra R . Single-use flexible ureteroscopes: comparative in vitro analysis of four scopes. Asian J Urol 2023;10:64–69. 36721687 10.1016/j.ajur.2022.02.001 PMC9875117

[61] Ventimiglia E Somani BK Traxer O . Flexible ureteroscopy: reuse? Or is single use the new direction? Curr Opin Urol 2020;30:113–119. 31815748 10.1097/MOU.0000000000000700

[62] Taguchi K Usawachintachit M Tzou DT . Micro-costing analysis demonstrates comparable costs for lithovue compared to reusable flexible fiberoptic ureteroscopes. J Endourol 2018;32:267–273. 29239227 10.1089/end.2017.0523

[63] Kramolowsky E McDowell Z Moore B . Cost analysis of flexible ureteroscope repairs: evaluation of 655 procedures in a community-based practice. J Endourol 2016;30:254–256. 26542761 10.1089/end.2015.0642

[64] Mager R Kurosch M Höfner T . Clinical outcomes and costs of reusable and single-use flexible ureterorenoscopes: a prospective cohort study. Urolithiasis 2018;46:587–593. 29356873 10.1007/s00240-018-1042-1

[65] Davis NF McGrath S Quinlan M . Carbon footprint in flexible ureteroscopy: a comparative study on the environmental impact of reusable and single-use ureteroscopes. J Endourol 2018;32:214–217. 29373918 10.1089/end.2018.0001

[66] Talyshinskii A Gauhar V Castellani D . Single use flexible ureteroscopes: a review of current technologies and cost effectiveness analysis. Curr Opin Urol 2024;34:110–115. 37962372 10.1097/MOU.0000000000001152

[67] Deng X Song L Xie D . A novel flexible ureteroscopy with intelligent control of renal pelvic pressure: an initial experience of 93 cases. J Endourol 2016;30:1067–1072. 27558001 10.1089/end.2015.0770

[68] De Coninck V Somani B Sener ET . Ureteral access sheaths and its use in the future: a comprehensive update based on a literature review. J Clin Med 2022;11:5128. 36079058 10.3390/jcm11175128 PMC9456781

[69] Chen H Qiu X Du C . The comparison study of flexible ureteroscopic suctioning lithotripsy with intelligent pressure control versus minimally invasive percutaneous suctioning nephrolithotomy in treating renal calculi of 2 to 3 cm in size. Surg Innov 2019;26:528–535. 31130072 10.1177/1553350619849782

[70] Zhu Z Cui Y Zeng F . Comparison of suctioning and traditional ureteral access sheath during flexible ureteroscopy in the treatment of renal stones. World J Urol 2019;37:921–929. 30120500 10.1007/s00345-018-2455-8

[71] Huang J Yang Y Xie H . Vacuum-assisted dedusting lithotripsy in the treatment of kidney and proximal ureteral stones less than 3 cm in size. World J Urol 2023;41:3097–3103. 37698634 10.1007/s00345-023-04595-6

[72] Ding J Su T Zhang X . Omni-directional (Flexible) ureteral access sheath: safety, efficacy, and initial experience report. J Endourol 2023;37:1184–1190. 37725564 10.1089/end.2023.0358

[73] Kaplan AG Lipkin ME Scales CD Jr . Use of ureteral access sheaths in ureteroscopy. Nat Rev Urol 2016;13:135–140. 26597613 10.1038/nrurol.2015.271

[74] De Coninck V Keller EX Rodríguez-Monsalve M . Systematic review of ureteral access sheaths: facts and myths. BJU Int 2018;122:959–969. 29752769 10.1111/bju.14389

[75] Hein S Wilhelm K Miernik A . Radiation exposure during retrograde intrarenal surgery (RIRS): a prospective multicenter evaluation. World J Urol 2021;39:217–224. 32200411 10.1007/s00345-020-03160-9 PMC7858553

[76] Müller PF Schlager D Hein S . Robotic stone surgery - Current state and future prospects: a systematic review. Arab J Urol 2018;16:357–364. 30140470 10.1016/j.aju.2017.09.004 PMC6104666

[77] Saglam R Muslumanoglu AY Tokatli Z . A new robot for flexible ureteroscopy: development and early clinical results (IDEAL stage 1-2b). Eur Urol 2014;66:1092–1100. 25059998 10.1016/j.eururo.2014.06.047

[78] Klein J Charalampogiannis N Fiedler M . Analysis of performance factors in 240 consecutive cases of robot-assisted flexible ureteroscopic stone treatment. J Robot Surg 2021;15:265–274. 32557097 10.1007/s11701-020-01103-5

[79] Shu X Hua P Wang S . Safety enhanced surgical robot for flexible ureteroscopy based on force feedback. Int J Med Robot 2022;18:e2410. 35439845 10.1002/rcs.2410

[80] De Coninck V Keller EX Somani B . Complications of ureteroscopy: a complete overview. World J Urol 2020;38:2147–2166. 31748953 10.1007/s00345-019-03012-1

[81] Chiu PK Chan CK Ma WK . Subcapsular hematoma after ureteroscopy and laser lithotripsy. J Endourol 2013;27:1115–1119. 23682955 10.1089/end.2013.0128

[82] Xu L Li G . Life-threatening subcapsular renal hematoma after flexible ureteroscopic laser lithotripsy: treatment with superselective renal arterial embolization. Urolithiasis 2013;41:449–451. 23800948 10.1007/s00240-013-0585-4

[83] Zhou L Cai X Li H . Effects of α-blockers, antimuscarinics, or combination therapy in relieving ureteral stent-related symptoms: a meta-analysis. J Endourol 2015;29:650–656. 25491604 10.1089/end.2014.0715 PMC4490592

[84] Corrales M Sierra A Doizi S . Risk of sepsis in retrograde intrarenal surgery: a systematic review of the literature. Eur Urol Open Sci 2022;44:84–91. 36071820 10.1016/j.euros.2022.08.008 PMC9442387

[85] Baboudjian M Gondran-Tellier B Abdallah R . Predictive risk factors of urinary tract infection following flexible ureteroscopy despite preoperative precautions to avoid infectious complications. World J Urol 2020;38:1253–1259. 31359106 10.1007/s00345-019-02891-8

[86] Bhojani N Miller LE Bhattacharyya S . Risk factors for urosepsis after ureteroscopy for stone disease: a systematic review with meta-analysis. J Endourol 2021;35:991–1000. 33544019 10.1089/end.2020.1133

[87] Sun J Xu J OuYang J . Risk factors of infectious complications following ureteroscopy: a systematic review and meta-analysis. Urol Int 2020;104:113–124. 31846966 10.1159/000504326

[88] Evans L Rhodes A Alhazzani W . Surviving sepsis campaign: international guidelines for management of sepsis and septic shock 2021. Intensive Care Med Nov 2021;47:1181–1247. 10.1007/s00134-021-06506-y PMC8486643 34599691

[89] Oczkowski S Alshamsi F Belley-Cote E . Surviving sepsis campaign guidelines 2021: highlights for the practicing clinician. Pol Arch Intern Med 2022;132:16290. 35791800 10.20452/pamw.16290

[90] Monnet X Lai C Teboul JL . How I personalize fluid therapy in septic shock? Crit Care 2023;27:123. 36964573 10.1186/s13054-023-04363-3 PMC10039545

[91] Zhu M Wang X Shi Z . Comparison between retrograde intrarenal surgery and percutaneous nephrolithotripsy in the management of renal stones: a meta-analysis. Exp Ther Med 2019;18:1366–1374. 31363376 10.3892/etm.2019.7710 PMC6614733

[92] Trelles Guzmán CR Mainez Rodríguez JA Aguado-Maestro I . 3D printed model for flexible ureteroscopy training, a low-cost option for surgical training. Actas Urol Esp (Engl Ed) 2022;46:16–21. 34844902 10.1016/j.acuroe.2021.07.009

[93] Chou DS Abdelshehid C Clayman RV . Comparison of results of virtual-reality simulator and training model for basic ureteroscopy training. J Endourol 2006;20:266–271. 16646655 10.1089/end.2006.20.266

[94] Cai JL Zhang Y Sun GF . Proficiency of virtual reality simulator training in flexible retrograde ureteroscopy renal stone management. Chin Med J (Engl) 2013;126:3940–3943. 24157162

